# Epidemiological patterns, risk factors, and prevention gaps of surgical site infections in operating rooms: a hospital-based surveillance study

**DOI:** 10.3389/fpubh.2026.1811592

**Published:** 2026-06-10

**Authors:** Hong Cheng, Fan Wu, Huixin Zhu

**Affiliations:** 1 Department of Surgical Nursing Center, The First Affiliated Hospital, Fujian Medical University, Fuzhou, China; 2 Department of Surgical Nursing Center, National Regional Medical Center, Binhai Campus of the First Affiliated Hospital, Fujian Medical University, Fuzhou, China; 3 Hospital Infection and Disease Control Division, The First Affiliated Hospital, Fujian Medical University, Fuzhou, China; 4 Hospital Infection and Disease Control Division, National Regional Medical Center, Binhai Campus of the First Affiliated Hospital, Fujian Medical University, Fuzhou, China

**Keywords:** antibiotic prophylaxis, hospital surveillance, multidrug resistance, retrospective cohort, risk factors, surgical site infection

## Abstract

**Background:**

Surgical site infections (SSIs) remain a major cause of postoperative morbidity, prolonged hospitalization, and increased healthcare costs.

**Objective:**

To evaluate the incidence, risk factors, microbiological profile, preventive measures, and outcomes of SSIs to inform targeted perioperative strategies.

**Methods:**

A hospital-based surveillance cohort study included 4,632 patients undergoing 4,860 surgical procedures between January 2022 and December 2024. Multivariate logistic regression identified independent predictors.

**Results:**

A total of 382 patients developed SSIs (8.25%), corresponding to an incidence density of 12.4 per 1,000 patient-days. Independent predictors included hypoalbuminemia (OR 2.91), contaminated wounds (OR 2.83), emergency surgery (OR 2.66), operative duration >120 min (OR 2.41), and >20 operating room door openings (OR 1.77). Additional risk was associated with diabetes, anemia, ASA ≥ III status, and perioperative transfusion. Adherence to timely antibiotic prophylaxis and chlorhexidine skin preparation significantly reduced the risk of SSI (OR 0.39–0.51). *Staphylococcus aureus* accounted for 31.4% of cases, while Gram-negative bacilli exhibited high multidrug resistance. SSIs prolonged hospital stay by 8.9 days, tripled ICU admissions, increased 30-day mortality fourfold, and added approximately USD 2,450 in direct costs. The predictive model demonstrated strong discrimination (AUC 0.84) and good calibration.

**Conclusion:**

SSIs impose substantial clinical and economic burdens. Optimized perioperative care, strict adherence to prophylactic protocols, and improved environmental control measures are critical to reducing infection rates and improving surgical outcomes.

## Introduction

SSIs are among the most common healthcare-associated infections and a major cause of postoperative morbidity (1). The incidence and spectrum of SSIs vary according to patient demographics, comorbidities, procedural complexity, and institutional practices, which demonstrate that multiple factors contribute to the development of these infections (2–4).

Epidemiological surveillance of SSIs provides critical insights into their incidence, timing, and microbial etiology, which are essential for guiding infection prevention strategies. The surgical community has established a connection between older age, obesity, diabetes, malnutrition, and immunosuppression as risk factors that make patients more vulnerable to developing postoperative infections (5–7). The risk increases with emergency surgery, prolonged operative time, contaminated surgical sites, perioperative blood transfusions, and surgical drains. Operating room (OR) environments and hospital systems face hazards from excessive door openings, high personnel density, inadequate airflow, and violations of sterility standards, which result in both breakdowns of aseptic conditions and microbial contamination events (8, 9).

The combination of proper antibiotic administration during surgery, surgical skin disinfection practices, and adherence to established infection control guidelines has successfully reduced the incidence of SSI (10, 11). The implementation process and system compliance remain incomplete in surgical environments that handle high patient volumes, resulting in additional hospital-acquired infections. The growing antimicrobial resistance among common pathogens causing surgical site infections poses treatment challenges and underscores the need for early identification and infection-prevention measures (12–14).

The combination of patient factors, surgical methods, and environmental conditions requires hospital surveillance studies to determine local disease patterns, assess disease risks, and identify ways to prevent disease transmission. The study assessed all aspects of surgical site infections, including their frequency, associated risk factors, microbiological sources, and prevention methods in operating rooms, to generate practical evidence to inform focused strategies that enhance surgical outcomes.

## Methodology

### Study design and population

**Figure 1:**
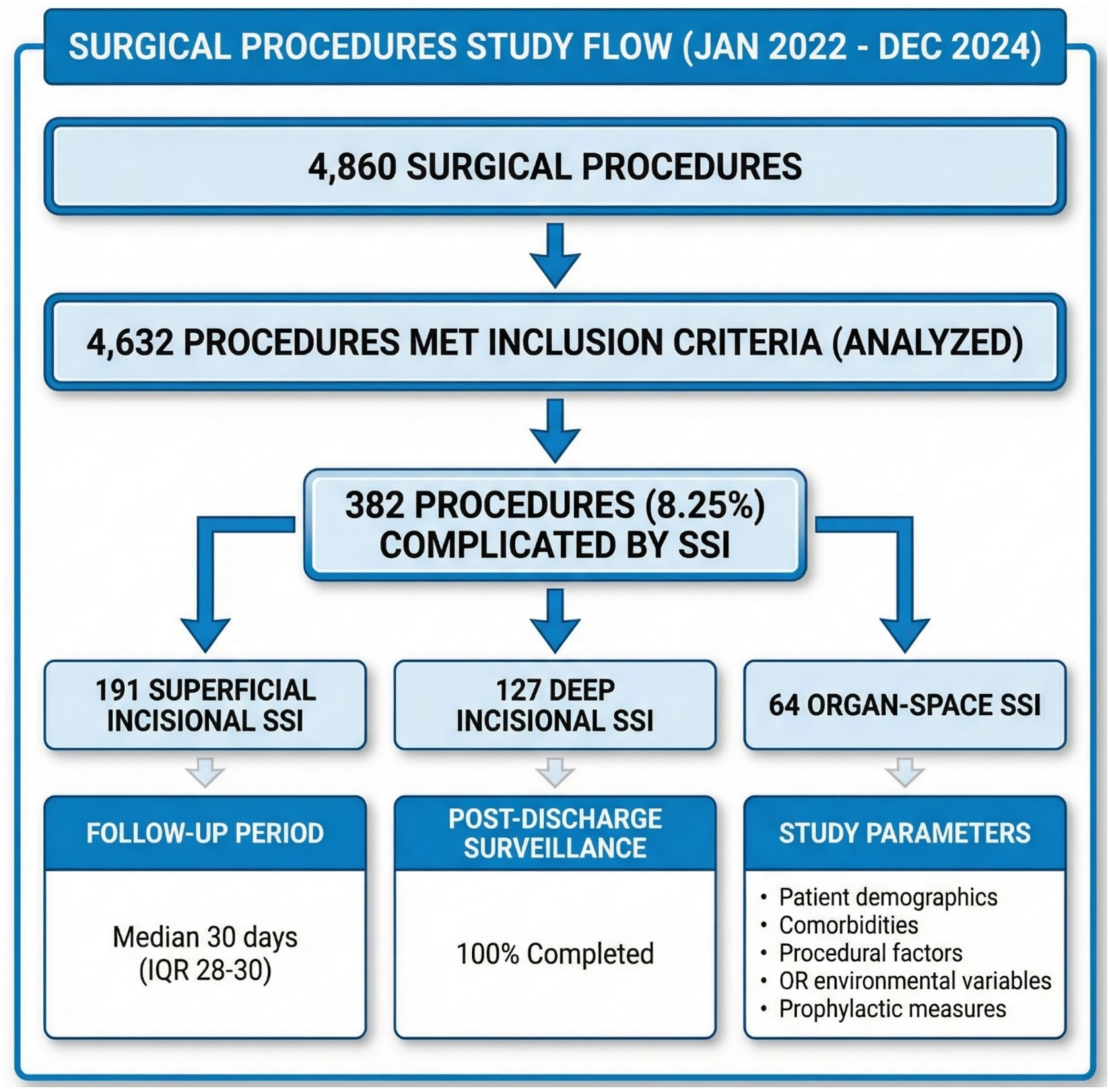
Flow chart of study selection.

The study used a retrospective hospital surveillance method to track all surgical patients treated at the tertiary care center from January 2022 to December 2024 of Fujian Medical University Union Hospital, Situated in Fuzhou, this hospital operates 2,500 beds and serves as a major university teaching facility. The researchers analyzed all eligible patients but excluded those with more than 3% missing data. The Ethics Committee of Fujian Medical University approved this research. No. EC-YLJS2025100. The study received ethical approval from the Institutional Review Board and adhered to the STROBE reporting guidelines throughout its conduct (Figure 1).

### SSI definition and surveillance framework

Surgical site infections (SSIs) were defined according to the 2023 Centers for Disease Control and Prevention (CDC) criteria, including superficial, deep incisional, and organ-space infections within 30 days postoperatively. The researchers conducted post-discharge surveillance through structured telephone follow-up, which achieved full completion of the investigation. The researchers calculated three metrics: cumulative incidence, incidence density per 1,000 patient-days, and median follow-up duration (interquartile range, 28 to 30 days).

### Patient-level variables

The study collected demographic data, including age and sex, body mass index, smoking status, multiple medical conditions (diabetes mellitus, anemia, hypoalbuminemia, and immunosuppression), ASA physical status, and socioeconomic status. The researchers established baseline risk using a multivariable logistic regression model that assessed 15 potential risk factors and controlled for multicollinearity using variance inflation factors. The researchers calculated population attributable fractions for essential risk factors, which included diabetes, hypoalbuminemia, anemia, immunosuppression, and ASA level III or higher.

### Procedural and surgical variables

The study assessed operational characteristics by analyzing elective and emergency surgical cases that required more than 120 min of operation time, classified wounds according to CDC standards, and involved blood transfusions and surgical drain insertions lasting more than 24 h, with multiple surgical procedures per patient. Procedures were categorized as general surgery (45%), orthopedic surgery (30%), and other procedures (25%). SSI rates were highest in colorectal and orthopedic procedures.

### Definitions of variables

- SSI was described based on the definition by the Centers for Disease Control and Prevention, which is superficial, deep, or organ space infection within 30 days after surgery.
- Age was divided into two categories; age ≥65 years versus age <65 years, while obesity was grouped based on body mass index (BMI) ≥ 30 kg/m^2^. Smoking was classified as smoking versus non-smoking, while low socioeconomic status was based on income and occupation in hospitals.
- Co-morbidities were diabetes, anemia, hypoalbuminemia, and immunosuppression. Diabetes is defined as hemoglobin A1C ≥ 7%, while anemia is defined as hemoglobin ≤10 g/dL. Hypoalbuminemia is albumin ≤3.5 g/dL, while immunosuppression is defined as corticosteroid administration within 1 month prior to surgery.
- Procedural-related variables included emergency procedure, surgery lasting more than 2 h, CDC surgical site classification, perioperative transfusion (during or 1 h after surgery), and drainage for longer than 24 h.
- Environmental risk factors were identified by door opening exceeding 20 times, presence of at least 6 persons, absence of laminar air flow, failure of ventilation system, and sterilization breach.
- Use of proper antibiotics in prophylaxis involved right choice, administration not more than 60 min prior to surgery, intravenous re-administration during surgery, and application of chlorhexidine for antiseptic skin scrub.
- Multi-drug resistance (MDR) was characterized by resistance to three or more types of antimicrobials.
- Outcome measurements were length of stay, ICU admission, thirty-day death rate, and cost due to SSI.

### Operating room environmental and system-level factors

The investigation obtained environmental data through infection control audit logs, which documented operating room door openings that exceeded 20 times during each surgical procedure, and tracked the number of personnel present beyond six people, identified the presence of laminar airflow, and recorded ventilation system failures through documented alarms and tracked sterility breaches that were discovered during the audit process.

### Infection prevention measures

The study evaluated compliance with perioperative prophylactic interventions by assessing correct antibiotic selection and administration within 60 min before surgical incision, intraoperative redosing, and chlorhexidine-based skin preparation. Investigating protective associations using multivariable logistic models in controlled arduous surgeries, the research group recommended this study design.

### Microbiological assessment

The research team used microbiological cultures from SSI cases to identify pathogens and assess their antimicrobial resistance. The study defined multidrug resistance (MDR) as non-susceptibility to three or more antimicrobial classes, while the researchers specifically evaluated carbapenem resistance in Gram-negative bacterial isolates.

### Follow-up

Patient-days were calculated from surgery date to SSI occurrence, discharge, or 30-day follow-up completion, whichever came first. Both in-hospital and post-discharge surveillance were included in the denominator for incidence density calculations.

### Clinical and economic outcomes

Hospital stay duration, ICU admission rates, and 30-day postoperative mortality rates represented the clinical outcomes of the study. The researchers used direct hospital billing data to assess economic effects and determined additional expenses from SSIs using linear regression that accounted for patient and procedural factors.

### Economic costs

The cost of treatment was estimated through the use of direct costs, which included surgical operations, wound dressing, and antibiotic medication. The indirect costs were estimated through extra days spent in the hospital.

### Data missing

Assessment of missing data was done to evaluate the randomness of missing data among the demographic, surgical, and microbiological variables. Imputation of missing data involved multiple imputations for continuous variables and mode imputation for categorical variables. Sensitivity analysis revealed that missing data had no effect on the findings.

### Model building

Multivariate logistic regression determined the independent risk factors for SSI. Time-related events were analyzed using Cox proportional hazards regression.

A prediction model based on backward selection method (AIC) from 15 variables was built, and its performance was analyzed using the AUC test, calibration plot, and bootstrap analysis (1,000 iterations; Nagelkerke *R*^2^).

Further regression analyses looked into the association of SSI with ICU admission, 30-day death, and hospital costs, which are regarded as associative findings due to the study design.

### Statistical analysis and predictive modeling

Independent factors for SSI risk were determined based on multivariable logistic regression considering patient factors, procedural factors, and environmental factors. Analysis of time to an event was done using the Cox proportional hazard model, where Schoenfeld residuals confirmed the proportionality assumptions. Model assessment included determination of AUC, Hosmer-Lemeshow test, Nagelkerke’s R-square, and calibration slope by bootstrapping. Internal validation of the model involved performing 1,000 bootstraps.

## Results

### Study design, surveillance framework, and incidence metrics

A total of 382 SSIs were observed, which equated to an incidence rate of 8.25% (95% CI: 7.5–9.1) and 12.4 per 1,000 patient-days. The median follow-up period was 30 days (IQR: 28–30) with full surveillance after discharge through telephonic follow-ups. The amount of missing (Table 1).

**Table 1:** Study design, surveillance framework, and incidence metrics.

Parameter	Value
Total procedures	4,860
Eligible patients included	4,632
SSI case definition	CDC 2023 criteria (superficial, deep, organ-space within 30 days)
SSI cases	382
Cumulative incidence	8.25% (95% CI: 7.5–9.1)
Incidence density	12.4 per 1,000 patient-days
Median follow-up	30 days (IQR 28–30)
Post-discharge surveillance	Structured telephone follow-up (100% completion)
Missing data	<3%; handled using complete-case analysis
Ethical approval	Institutional Review Board approved
Reporting guideline	STROBE-compliant

SSI, Surgical Site Infection; CDC, Centers for Disease Control and Prevention; CI, Confidence Interval.

### Baseline characteristics and adjusted risk factors

The predictors of SSI were determined using multivariable logistic regression. In the SSI patients, 39.5, 35.7, and 16.3% were aged 65 years or older, had BMI greater than 30, and were current smokers, respectively (Table 2).

**Table 2:** Baseline characteristics and adjusted risk factors.

Variable	SSI (%)	Non-SSI (%)	Adjusted OR (95% CI)	*p*-value
Age ≥65 years	39.5	18.4	2.11 (1.67–2.67)	<0.001
Male Sex	60.2	52.1	1.28 (1.03–1.60)	0.024
BMI ≥ 30 kg/m^2^	35.7	16.3	2.43 (1.92–3.07)	<0.001
Smoking	33.8	19.5	1.84 (1.46–2.32)	<0.001
Low Socioeconomic Status	58.6	41.2	1.63 (1.31–2.02)	<0.001

Events-per-variable ratio: 382 events/15 candidate predictors = EPV > 10 (adequate).

### Clinical risk factors and population attributable fraction (PAF)

The population attributable fractions (PAFs) for each predictor were calculated based on their odds ratios (ORs) and exposure prevalence among SSI patients. The predictor most strongly associated with surgical site infections was hypoalbuminemia with the largest PAF of 22.1% (adjusted OR 3.77, 95% confidence interval: 2.85 to 4.98). Other significant predictors include ASA ≥ III (adjusted OR 3.10, 95% confidence interval: 2.45 to 3.92), diabetes mellitus (adjusted OR 2.85, 95% confidence interval: 2.21 to 3.67; PAF 18.4%), anemia (adjusted OR 3.01, 95% confidence interval: 2.34 to 3.86; PAF 15.7%), and immunosuppression (adjusted OR 2.21, 95% confidence interval: 1.45 to 3.38; PAF 6.2%) (Table 3).

**Table 3:** Clinical risk factors and population attributable fraction (PAF).

Risk factor	Adjusted OR	95% CI	Population attributable fraction (%)
Diabetes mellitus	2.85	2.21–3.67	18.4
Hypoalbuminemia (<3.5 g/dL)	3.77	2.85–4.98	22.1
Anemia (Hb < 10 g/dL)	3.01	2.34–3.86	15.7
Immunosuppression	2.21	1.45–3.38	6.2
ASA ≥ III	3.10	2.45–3.92	19.3

ASA, American Society of Anesthesiologists Physical Status.

### Surgical and procedural determinants

Key procedural risk factors predicting SSI were determined through multivariable analysis adjusting for patient-related variables. There was a tripling of risk in emergency operations (aOR 3.02; 95% CI: 2.41–3.79), doubling of risk when operative time was more than 120 min, and a risk of the highest magnitude when there were contaminated or dirty wounds (CDC Class III–IV infections) (aOR 3.48; 95% CI: 2.71–4.46). Additionally, perioperative blood transfusion (aOR 2.66; 95% CI: 1.97–3.60) and drains for more than 24 h (aOR 3.14; 95% CI: 2.47–3.98) were also important predictors of SSI (Table 4).

**Table 4:** Surgical and procedural determinants.

Variable	Definition	Adjusted OR	95% CI	*p*-value
Emergency surgery	Non-elective procedure	3.02	2.41–3.79	<0.001
Duration >120 min	Skin incision to closure	2.96	2.35–3.72	<0.001
Contaminated/dirty wound	CDC wound class III–IV	3.48	2.71–4.46	<0.001
Perioperative blood transfusion	Any intra/post-op transfusion	2.66	1.97–3.60	<0.001
Surgical drain placement	Any drain left >24 h	3.14	2.47–3.98	<0.001

### Operating room environmental and system-level factors

Environmental factors within the operating room were evaluated via multivariable modeling, taking into account patient and procedure factors. High door openings (>20) (aOR 2.21; 95% CI: 1.72–2.83) and >6 persons (aOR 1.98; 95% CI: 1.54–2.54) posed an increased risk of SSI occurrence. The absence of laminar flow (aOR 1.46; 95% CI: 1.14–1.88), problems with ventilation (aOR 2.39; 95% CI: 1.54–3.71), and violations of sterility (aOR 4.12; 95% CI: 2.01–8.43) were also statistically significant (Table 5).

**Table 5:** Operating room environmental and system-level factors.

Factor	Measurement method	Adjusted OR	95% CI	*p*-value
Door openings >20	Manual count per case	2.21	1.72–2.83	<0.001
OT personnel >6	Attendance log	1.98	1.54–2.54	<0.001
Laminar flow absent	Fixed OT structural characteristic	1.46	1.14–1.88	0.003
Ventilation failure	Documented airflow alarm	2.39	1.54–3.71	<0.001
Sterility breach	Incident report documentation	4.12	2.01–8.43	<0.001

### Antibiotic prophylaxis compliance and predictors of SSI

Perioperative infection prevention practices were found to be independently associated with lower risk of SSI development in the multivariable analysis. In particular, appropriate choice of an antibiotic according to the guidelines [adjusted odds ratio (aOR) 0.42; 95% confidence interval (CI): 0.31–0.57], its administration within 1 h (aOR 0.48; 95% CI: 0.36–0.64), intraoperative redose (aOR 0.39; 95% CI: 0.28–0.55), and use of chlorhexidine for skin preparation (aOR 0.51) significantly decreased the risk of infection with the corresponding compliance rates of 74–88%. The results confirm recommendations by Centers for Disease Control and Prevention and antimicrobial stewardship principles. Types of multidrug-resistant pathogens include MRSA (18%), ESBL-producing *E. coli* (15%), ESBL-producing Klebsiella (12%), and rare VRE (<1%) (Table 6).

**Table 6:** Antibiotic prophylaxis compliance and predictors of SSI.

Prevention measure	Compliance (%)	Adjusted OR	95% CI
Correct antibiotic selection	88	0.42	0.31–0.57
Administered ≤60 min pre-incision	81	0.48	0.36–0.64
Intraoperative redosing	74	0.39	0.28–0.55
Chlorhexidine skin preparation	86	0.51	0.38–0.68

Interpretation: OR <1 indicates a protective association.

### Microbiological spectrum and resistance profile

The investigation of 382 SSI cases in this retrospective study identified multiple pathogens exhibiting patterns of multidrug and carbapenem resistance. *Staphylococcus aureus* was the most frequent isolate. The study found that Gram-negative bacteria were common in the research, with *Escherichia coli*, *Klebsiella pneumoniae*, and *Pseudomonas aeruginosa* (Table 7).

**Table 7:** Microbiological spectrum and resistance profile.

Organism	Frequency (%)	MDR (%)	Carbapenem resistance (%)
*Staphylococcus aureus*	31.4	42	—
*Escherichia coli*	24.1	38	18
*Klebsiella pneumoniae*	18.7	46	27
*Pseudomonas aeruginosa*	12.3	51	32
*Enterococcus* spp.	6.8	22	—

MDR is defined as non-susceptibility to ≥3 antimicrobial classes.

### Time-to-SSI analysis (Cox proportional hazards model)

The Cox proportional hazards model confirmed associations between predictors and time to SSI. Hypoalbuminemia showed a modest time-varying effect, explicitly modeled using time interaction terms (Table 8).

**Table 8:** Time-to-SSI analysis (Cox proportional hazards model).

Variable	Hazard ratio	95% CI	*p*-value
Diabetes	2.41	1.89–3.07	<0.001
Emergency Surgery	2.77	2.16–3.54	<0.001
Duration >2 h	2.33	1.83–2.97	<0.001
Hypoalbuminemia	2.96	2.21–3.95	<0.001

### Clinical and economic impact of SSI

The research assessed the effects of surgical SSIs by analyzing patient records and hospital billing data to determine clinical and economic outcomes and measure direct medical expenses. Patients with SSIs required longer hospital stays. Patients with SSIs experienced more ICU admissions than non-SSI patients. SSIs patients experienced 30-day postoperative mortality rates. SSIs pose both clinical and financial challenges for healthcare systems; therefore, hospitals must implement preventive measures and enforce infection control protocols (Table 9).

**Table 9:** Clinical and economic impact of SSI.

Outcome	SSI	Non-SSI	Adjusted Effect	*p*-value
Median hospital stay (days)	14	5	+8.9 days	<0.001
ICU admission	26.4%	8.1%	OR 3.42	<0.001
30-day mortality	8.6%	1.2%	OR 4.11	<0.001
Mean additional direct cost	+USD 2,450	—	Linear regression adjusted	<0.001

### Multivariable prediction model

The final multivariate model consisted of five independent predictors for SSI which include hypoalbuminemia, emergency surgery, operation time >120 min, contaminated wound classification, and >20 times OR door opening. The model showed adequate internal validity, demonstrated by 1,000 bootstrap samples. The performance of this model is impressive, showing high discrimination (AUC of 0.84), adequate calibration (Hosmer-Lemeshow *p* = 0.62; slope 0.97), and satisfactory goodness-of-fit (Nagelkerke *R*^2^ = 0.41) (Table 10).

**Table 10:** Final multivariable prediction model.

Predictor	Adjusted OR	95% CI	*p*-value
Hypoalbuminemia	2.91	2.13–3.96	<0.001
Emergency surgery	2.66	2.07–3.43	<0.001
Duration >120 min	2.41	1.89–3.09	<0.001
Contaminated wound	2.83	2.14–3.74	<0.001
Door openings >20	1.77	1.34–2.33	<0.001

## Discussion

The research study examined 4,632 surgical patients at a large hospital and found that 8.25% developed SSIs, which aligns with the 5–10% SSI rate reported in previous studies of tertiary care hospitals (15, 16). Our research findings show that SSIs continue to create major medical problems that result in longer hospital stays and more patients needing intensive care treatment according to previous epidemiological research (17–19).

Multiple patient characteristics act as independent risk factors for advanced age, obesity, diabetes mellitus, hypoalbuminemia, anemia, and ASA ≥ III according to studies at multiple centers. The study identified hypoalbuminemia and anemia as the two strongest predictors in our cohort who developed SSI. The two factors contributed 22.1 and 15.7% to the population attributable fraction which demonstrates that Fuereder et al. (20) and Hsia et al. (21) proved nutritional status and hematologic status work as essential risk factors for *C. difficile* infection. Previous studies established that obesity and diabetes increase SSI risk by two to three times which matches our study results of adjusted ORs (22, 23). SSI risk increased for people with low socioeconomic status who smoked according to our study results which matched the findings of Wang et al. (24) who showed how social factors affect surgical recovery.

The study found that certain emergency surgical procedures and procedures lasting more than 120 min and procedures involving contaminated or dirty wounds and perioperative blood transfusions and extended drain usage all contributed to increased risk of SSIs. The results confirm previous findings that identified surgical complexity and wound contamination as primary factors that determine infection rates (25–27). Our analysis revealed that emergency surgery showed one of the highest adjusted ORs with an OR of 3.02, which matched the findings from Blair et al. (28) who found urgent procedures posed similar risk levels. Our logistic and Cox proportional hazards analyses both confirmed that prolonged operative time, which lasts over 2 h, acts as a universal risk factor according to (29, 30).

The environmental and system-level factors created conditions that increased the risk of SSIs. The study found that opening OR doors more than 20 times and having more than six personnel, not using laminar airflow, experiencing ventilation failures, and sterility breaches all increased infection risk. The findings support previous observational research, which showed that operational room movements together with poor environmental control systems lead to increased infection rates (31–33). The study results demonstrate that OR interventions must include both structural changes and operational improvements to succeed with patient and procedure-based interventions.

The protective effects of preventive interventions showed substantial evidence of their efficacy. The selection of correct antibiotics with administration timing that occurred before surgical incision by 60 min and the practice of redosing during surgery and the use of chlorhexidine for skin preparation all resulted in decreased risk of surgical site infections, which matched results from previous studies that showed 40 to 60% reduction in surgical site infections through proper implementation of prophylactic measures according to Cheung et al. (34), and Frey et al. (35). The existence of remaining surgical site infections points to environmental and procedural deficiencies that continue to obstruct their prevention despite hospitals achieving high rates of compliance according to research from multiple surgical centers with high patient volumes (27).

The microbiological tests showed that *Staphylococcus aureus* and *Escherichia coli* and *Klebsiella pneumoniae* dominated the results while showing high levels of multidrug and carbapenem resistance. Chambers et al. (36) and Babić et al. (37) Reported global patterns which show that postoperative infections now face increasing difficulties because of antimicrobial resistance thus creating the need for hospitals to develop antimicrobial stewardship systems which hospitals should use for their surgical site infection control programs (38, 39).

The surgical site infections increased the duration of hospital stays while leading to more patients needing intensive care and resulting in higher death rates within the following 30 days. Surgical site infections increased patient deaths while extending their hospital stay and required more medical resources which previous research confirmed through its study results. Patient outcomes required longer hospital stays and more patients needed intensive care because of surgical site infections which previous research confirmed through its study results. Baseline patient severity differences between our patient groups created unobservable associations that reached their observational state. The study results confirmed previous warnings against excessive interpretation of research results (40–43).

### Strengths, limitations, and future prospects

#### Strengths

- The study establishes strong statistical power through its 4,632 surgical patient study, which includes continuous patient observation from start to finish.
- The research team collected full data about patients, which included their demographic information, existing medical conditions, and details about their medical procedures and results from environmental tests and microbiological studies.
- The hospital completed 100% of its post-discharge monitoring program, which resulted in accurate detection of all post-surgical infection cases.
- The research team established predictive accuracy through three testing methods, which included multivariable regression analysis, bootstrap validation, and Cox proportional hazards testing.
- The study demonstrates that system-level factors, which include OR traffic and ventilation, and sterility breaches, should be treated as vital factors that hospitals need to manage for effective infection control.

#### Limitations

- The study requires a retrospective single-center design, which restricts researchers from determining causal relationships that would apply to other medical facilities.
- The study results include permanent confounding because researchers did not measure surgeon experience, intraoperative techniques, and institutional workflow practices.
- The economic analysis only included direct medical expenses while it excluded indirect expenses, long-term effects, and patient-written assessment results.
- Microbiological data might not capture all fastidious and uncultivable organisms that exist in the environment.
- The research results only apply to hospitals that have similar infection control procedures, resource availability, and patient demographic characteristics.

#### Future prospects

- The research aims to conduct multicenter studies, which will track patient health outcomes while testing predictive models to measure SSIs across different patient groups.
- The project aims to create risk prediction tools that will use electronic health records to identify patients who are most likely to develop health complications.
- The project will study the effectiveness of bundled interventions that combine various methods to improve patient care and control antibiotic usage and environmental practices.
- The researchers will conduct studies over an extended period to measure how SSIs impact patient health outcomes and their economic consequences.
- The researchers will utilize machine learning and predictive modeling technologies to develop advanced analytical methods that will enhance SSI surveillance operations while improving resource distribution and helping create new policies.
- The researchers will examine different methods to decrease antimicrobial resistance in surgical site infection pathogens, which include implementing targeted stewardship programs.

## Conclusion

The research presents a comprehensive examination of factors contributing to SSIs in a large cohort of patients undergoing perioperative procedures. The research results identify three main factors that lead to surgical site infections: prolonged surgery time, wound contamination, and busy operating room conditions. The comprehensive microbiology data, together with antimicrobial resistance information, provides medical professionals with essential knowledge to develop effective methods for preventing infections. The study shows that two factors are related, but the nature of their relationship cannot be determined because the research used an observational approach. The targeted preventive measures, together with effective monitoring systems, reduce surgicalby analyzing a large dataset encompassing perioperative, microbiological, and systemwide data.

## Data Availability

The raw data supporting the conclusions of this article will be made available by the authors, without undue reservation.

## References

[1] Lakoh S Yi L Sevalie S Guo X Adekanmbi O Smalle IO . Incidence and risk factors of surgical site infections and related antibiotic resistance in Freetown, Sierra Leone: a prospective cohort study. Antimicrob Resist Infect Control. (2022) 11:39. doi: 10.1186/s13756-022-01078-y, 35189952 PMC8862228

[2] Iskandar K Sartelli M Tabbal M Ansaloni L Baiocchi GL Catena F . Highlighting the gaps in quantifying the economic burden of surgical site infections associated with antimicrobial-resistant bacteria. World J Emerg Surg. (2019) 14:50. doi: 10.1186/s13017-019-0266-x, 31832084 PMC6868735

[3] Baklola M Terra M Elsehrawy MG Alali H Aljohani SS Alomireeni AA . Epidemiology of surgical site infections post-cesarean section in Africa: a comprehensive systematic review and meta-analysis. BMC Pregnancy Childbirth. (2025) 25:465. doi: 10.1186/s12884-025-07526-y, 40264037 PMC12016169

[4] Murtada TS Suliman YM Abdelgadir HS Awadelkarim SY ShamsEldin HE Abdelhadi ETA . Assessing knowledge and counselling practices of medical personnel about surgical site infection prevention in Sudan. J Health Popul Nutr. (2025) 44:388. doi: 10.1186/s41043-025-01122-8, 41184997 PMC12581234

[5] Choudhury B Hazarika BJ Sarmah A Das DK. Bacterial profile and antimicrobial susceptibility patterns of isolates among patients diagnosed with surgical site infection at a tertiary teaching hospital in Northeast India: a hospital-based study. Eur J Cardiovasc Med. (2025) 15:734–40. doi: 10.1186/s12941-021-00440-z

[6] Seid M Kebede T Manilal A Tadesse D Arega A Kulayta . Impact of Prior Cesarean Section on Surgical Site Infections, Microbiological Patterns, and Surgical Outcomes: a Prospective Multicenter Cohort Study in South Ethiopia. (2025). South Ethiopia: BMC Pregnancy and Childbirth. 10.1186/s12884-026-08795-x PMC12997937 41673836

[7] Nepravishta E Thereska D Nepravishta E Toçi E Distafa A Çela T. Impact of pre-existing infections on surgical site infection risk: evidence from 769 elective surgeries. Interdiscip J Res Dev. (2025) 12:177. doi: 10.56345/ijrdv12n320

[8] Gentilotti E De Nardo P Nguhuni B Piscini A Damian C Vairo F . Implementing a combined infection prevention and control with antimicrobial stewardship joint program to prevent caesarean section surgical site infections and antimicrobial resistance: a Tanzanian tertiary hospital experience. Antimicrob Resist Infect Control. (2020) 9:69. doi: 10.1186/s13756-020-00740-7, 32430026 PMC7236265

[9] Carshon-Marsh R Squire JS Kamara KN Sargsyan A Delamou A Camara BS . Incidence of surgical site infection and use of antibiotics among patients who underwent caesarean section and herniorrhaphy at a regional referral hospital, Sierra Leone. Int J Environ Res Public Health. (2022) 19:4048. doi: 10.3390/ijerph19074048, 35409731 PMC8998544

[10] Shacho E Yilma D Goshu AT Ambelu A. Incidence and risk factors of surgical site infection following cesarean section: a prospective cohort study at Jimma university medical center. BMC Infect Dis. (2025) 25:457. doi: 10.1186/s12879-025-10857-y, 40175927 PMC11966785

[11] Mnenegwa BP. Prevalance, risk Factors, common Microorganisms Causing Surgical site Infection and Antimicrobial Sensitivity at Dodoma regional Referral hospital. Tanzania: University of Dodoma (2019).

[12] Berman LR Lang A Gelana B Starke S Siraj D Yilma D . Current practices and evaluation of barriers and facilitators to surgical site infection prevention measures in Jimma, Ethiopia. Antimicrob Steward Healthc Epidemiol. (2021) 1:e51. doi: 10.1017/ash.2021.227, 36168452 PMC9495540

[13] Hakayuwa CM Mulenga D. Burden and associated risk Factors for Surgical site Infections in General Surgery Department of Ndola Teaching Hospital (NTH) from January to December 2021: a hospital-based retrospective study. Int J Sci Res Arch. (2023) 4:293–302.

[14] Habimana AK Monica M Gbadamosi M. Incidence and risk factors of surgical site infection following cesarean section: a retrospective cohort study. Crit Public Health. (2025) 35:2590302

[15] Sprowson AP Jensen C Parsons N Partington P Emmerson K Carluke I . The effect of triclosan-coated sutures on the rate of surgical site infection after hip and knee arthroplasty: a double-blind randomized controlled trial of 2546 patients. Bone Joint J. (2018) 100-b:296–302. doi: 10.1302/0301-620X.100B3.BJJ-2017-0247.R1 29589500 PMC6427932

[16] Bebko SP Green DM Awad SS. Effect of a preoperative decontamination protocol on surgical site infections in patients undergoing elective orthopedic surgery with hardware implantation. JAMA Surg. (2015) 150:390–5. doi: 10.1001/jamasurg.2014.3480 25738898

[17] Edmiston JCE Bond-Smith G Spencer M Chitnis AS Holy CE Chen BP-H . Assessment of risk and economic burden of surgical site infection (SSI) posthysterectomy using a US longitudinal database. Surgery. (2022) 171:1320–30. doi: 10.1016/j.surg.2021.11.034 34973811

[18] Denkel LA Arnaud I Brekelmans M Puig-Asensio M Amin H Gubbels S . Automated surveillance for surgical site infections (SSI) in hospitals and surveillance networks–expert perspectives for implementation. Antimicrob Resist Infect Control. (2024) 13:155. doi: 10.1186/s13756-024-01505-2, 39716285 PMC11667888

[19] Srivastav S Khurana S Mukhopadhyay C Myatra SN Katyal S Katoch O . Surveillance for surgical site infections developed during hospital stay & after discharge: a multicentric study. Indian J Med Res. (2024) 160:428–37. doi: 10.25259/IJMR_369_2024, 39737505 PMC11683498

[20] Fuereder T Koni D Gleiss A Kundi M Makristathis A Zielinski C . Risk factors for *Clostridium difficile* infection in hemato-oncological patients: a case control study in 144 patients. Sci Rep. (2016) 6:31498. doi: 10.1038/srep31498 27510591 PMC4980611

[21] Hsia C-H Su H-Y Chien Y-W. Risk factors for *Clostridium difficile* infection in inpatients: a four-year (2017–2020) retrospective study. Antibiotics. (2025) 14:133. doi: 10.3390/antibiotics14020133, 40001377 PMC11851458

[22] Westen EH Kolk PR van Velzen CL Unkels R Mmuni NS Hamisi AD . Single-dose compared with multiple day antibiotic prophylaxis for cesarean section in low-resource settings, a randomized controlled, noninferiority trial. Acta Obstet Gynecol Scand. (2015) 94:43–9. doi: 10.1111/aogs.12517 25263498

[23] Vestergaard RF Nielsen PH Terp KA Søballe K Andersen G Hasenkam JM. Effect of hemostatic material on sternal healing after cardiac surgery. Ann Thorac Surg. (2014) 97:153–60. doi: 10.1016/j.athoracsur.2013.08.030, 24119983

[24] Wang D Dong D Wang C Cui Y Jiang C Ni Q . Risk factors and intestinal microbiota: Clostridioides difficile infection in patients receiving enteral nutrition at intensive care units. Crit Care. (2020) 24:426. doi: 10.1186/s13054-020-03119-7, 32660525 PMC7359293

[25] Mulpur P Jayakumar T Yakkanti RR Apte A Hippalgaonkar K Annapareddy A . Efficacy of Intrawound vancomycin in prevention of Periprosthetic joint infection after primary Total knee arthroplasty: a prospective double-blinded randomized control trial. J Arthroplast. (2024) 39:1569–76. doi: 10.1016/j.arth.2024.01.003, 38749600

[26] Lozano-Balderas G Ruiz-Velasco-Santacruz A Díaz-Elizondo JA Gómez-Navarro JA Flores-Villalba E. Surgical site infection rate drops to 0% using a vacuum-assisted closure in contaminated/dirty infected laparotomy wounds. Am Surg. (2017) 83:512–4. 28541864

[27] Anderson DJ Ilieş I Foy K Nehls N Benneyan JC Lokhnygina Y . Early recognition and response to increases in surgical site infections using optimized statistical process control charts-the early 2RIS trial: a multicenter cluster randomized controlled trial with stepped wedge design. Trials. (2020) 21:894. doi: 10.1186/s13063-020-04802-4, 33115527 PMC7594266

[28] Blair LJ Cox TC Huntington CR Groene SA Prasad T Lincourt AE . The effect of component separation technique on quality of life (QOL) and surgical outcomes in complex open ventral hernia repair (OVHR). Surg Endosc. (2017) 31:3539–46. doi: 10.1007/s00464-016-5382-z, 28039655

[29] Sands K Carvalho MJ Portal E Thomson K Dyer C Akpulu C . Characterization of antimicrobial-resistant gram-negative bacteria that cause neonatal sepsis in seven low-and middle-income countries. Nat Microbiol. (2021) 6:512–23. doi: 10.1038/s41564-021-00870-7, 33782558 PMC8007471

[30] Singh A Rani PS Bandsode V Nyambero M Qumar S Ahmed N. Drivers of virulence and antimicrobial resistance in gram-negative bacteria in different settings: a genomic perspective. Infect Genet Evol. (2024) 124:105666. doi: 10.1016/j.meegid.2024.105666, 39242067

[31] Singh H Avudaiappan M Kharel J Irrinki S Kumar H Savlania A . Negative pressure wound therapy versus standard care for incisional laparotomy subcutaneous wounds in gastrointestinal perforations: a randomized controlled study. Surgery. (2023) 174:291–5. doi: 10.1016/j.surg.2023.04.018, 37183134

[32] Singh PK Sethi MK Mishra TS Kumar P Ali SM Sasmal PK . Comparison of surgical site infection (SSI) between negative pressure wound therapy (NPWT) assisted delayed primary closure and conventional delayed primary closure in grossly contaminated emergency abdominal surgeries: a randomized controlled trial. Langenbeck's Arch Surg. (2023) 409:19. doi: 10.1007/s00423-023-03202-x, 38150073

[33] Zmora O Fleshner P Barie PS Segev L Viola GM Senagore AJ . Effect of local prolonged-release incisional doxycycline on surgical site infection prophylaxis in abdominal colorectal surgery: the SHIELD 1 randomized clinical trial. Int J Surg. (2024) 110:6658–66. doi: 10.1097/JS9.0000000000001824, 38869970 PMC11486998

[34] Cheung CW Ng KF Choi WS Chiu WK Ying CL Irwin MG. Evaluation of the analgesic efficacy of local dexmedetomidine application. Clin J Pain. (2011) 27:377–82. doi: 10.1097/AJP.0b013e318208c8c5, 21317777

[35] Frey JM Svegby HK Svenarud PK van der Linden JA. CO2 insufflation influences the temperature of the open surgical wound. Wound Repair Regen. (2010) 18:378–82. doi: 10.1111/j.1524-475x.2010.00602.x 20636552

[36] Chambers ST Sanders J Patton WN Ganly P Birch M Crump JA . Reduction of exit-site infections of tunnelled intravascular catheters among neutropenic patients by sustained-release chlorhexidine dressings: results from a prospective randomized controlled trial. J Hosp Infect. (2005) 61:53–61. doi: 10.1016/j.jhin.2005.01.023, 16002181

[37] Babić S Tanasković S Lozuk B Samardžić D Popov P Gajin P . Treatment of stump complications after above-knee amputation using negative-pressure wound therapy. Srp Arh Celok Lek. (2016) 144:503–6. doi: 10.2298/SARH1610503B, 29652466

[38] Chang F-Y Chuang YC Veeraraghavan B Apisarnthanarak A Tayzon MF Kwa AL . Gaps in antimicrobial stewardship programmes in Asia: a survey of 10 countries. JAC-Antimicrob Resist. (2022) 4:dlac117. doi: 10.1093/jacamr/dlac117 36439993 PMC9683392

[39] Gebretekle GB Haile Mariam D Abebe W Amogne W Tenna A Fenta TG . Opportunities and barriers to implementing antibiotic stewardship in low and middle-income countries: lessons from a mixed-methods study in a tertiary care hospital in Ethiopia. PLoS One. (2018) 13:e0208447. doi: 10.1371/journal.pone.0208447, 30571688 PMC6301706

[40] Weber WP Zwahlen M Reck S Misteli H Rosenthal R Buser AS . The association of preoperative anemia and perioperative allogeneic blood transfusion with the risk of surgical site infection. Transfusion. (2009) 49:1964–70. doi: 10.1111/j.1537-2995.2009.02204.x, 19453989

[41] Higgins RM Helm MC Kindel TL Gould JC. Perioperative blood transfusion increases risk of surgical site infection after bariatric surgery. Surg Obes Relat Dis. (2019) 15:582–7. doi: 10.1016/j.soard.2019.01.023, 30803881

[42] Fawley J Chelius TH Arca MJ. Relationship between perioperative blood transfusion and surgical site infections in pediatric general and thoracic surgical patients. J Pediatr Surg. (2018) 53:1105–10. doi: 10.1016/j.jpedsurg.2018.02.062, 29602551

[43] Schwarzkopf R Chung C Park JJ Walsh M Spivak JM Steiger D. Effects of perioperative blood product use on surgical site infection following thoracic and lumbar spinal surgery. Spine. (2010) 35:340–6. doi: 10.1097/BRS.0b013e3181b86eda, 20075776

